# Learning from the Experiences of Autistic Professionals Working in Health and Education

**DOI:** 10.1089/aut.2024.0168

**Published:** 2025-01-20

**Authors:** Eleanor Curnow, Donald Maciver, Lorna Johnston, Mairead Murray, Victoria Johnstone-Cooke, Izy Utley, Natalie Jenkins, Tamsin Meff, Joshua Muggleton, Marion Rutherford

**Affiliations:** 1 School of Health Sciences, Queen Margaret University, Edinburgh, United Kingdom of Great Britain and Northern Ireland.; 2 Clinical Psychology Dept, NHS Fife, Lynebank Hospital, Dunfermline, United Kingdom of Great Britain and Northern Ireland.

**Keywords:** thematic analysis, employment, career progression, autism, discrimination, neuro-inclusion

## Abstract

**Background:**

This study aimed to explore the experiences of autistic professionals working in the public sector in Scotland and investigate the factors that supported them in achieving employment in their chosen career.

**Methods:**

We interviewed 34 autistic adults employed in professional roles in the health and education sectors in Scotland about their successes and challenges during training, recruitment, and employment. Interview conversations of 60–90-minute duration were transcribed verbatim. The research team, which included autistic and non-autistic researchers, conducted inductive thematic analysis.

**Results:**

Resultant themes included complexity of disclosure; navigating differences in social communication and across employment stages; and control of the environment. Autistic professionals face inequalities and unsupportive environments. Participants described multifaceted decision-making processes behind choices to disclose or withhold their autism diagnosis, which could determine their right to workplace accommodations or provoke unfavorable treatment. The styles of communication preferred by colleagues could engender misunderstanding and lead to challenges negotiating social situations, recruitment processes, and organizational culture. Participants’ needs and preferences for predictability and routine required them to use strategies to overcome the negative impacts of suboptimal social and physical environments.

**Conclusion:**

Findings confirm the importance of acceptance and inclusion and demonstrate that there is a need for culture change within public sector education and health workplaces to improve accessibility. Applying minor changes to the environment and individual communication styles can enhance workplace conditions for autistic employees.

**Community Brief:**

**Why is this an important issue?**

Fewer autistic people are employed in skilled jobs than we would expect, and autistic graduates are less likely to be employed than graduates with any other disability. We need research to understand what helps autistic people in skilled work and career development, for example, through promotion. It is important that autistic people can follow their chosen career paths without risk to their health.

**What was the purpose of this study?**

We wanted to know more about the experiences of autistic people employed in public sector professional roles in Scotland. We wanted to find out about work, training, and recruitment from their point-of-view. We wanted to understand what worked well, and what employers can improve.

**What did the researchers do?**

We interviewed 34 autistic people working in professional roles in health or education in Scotland (including nurses, doctors, teachers, occupational therapists, clinical scientists, and radiographers). We thought about what people told us to help us understand their experiences.

**What were the results of the study?**

Autistic professionals said that the decision to share their autism diagnosis at work was complicated. Before deciding to share this information, they thought about the risk of discrimination or stigma. They thought about how this might affect their access to support at work, and their wish to raise awareness of autism by acting as a role model. Sometimes colleagues communicated in ways which led to misunderstandings. Autistic employees told us that the habits and ways of working at their workplace did not suit them. They reported that work environments did not support their needs. This caused them stress and meant that they needed to find ways to stay in employment and look after their health.

**What do these findings add to what is already known?**

We already know that autistic employees face discrimination and stress in the workplace. Our research confirms that there is a need for changes to support autistic professionals. The untrue or unfair views that colleagues have about autism (and other distinctions such as age, ethnicity, race, sex, social and economic status, or gender) create discrimination and prevent people receiving support at work or progressing in their career.

**What are the potential weaknesses in the study?**

People who took part in this research were all professionals working in Scotland and were mainly White, female graduates. They had all been working for at least 2 years. This means that the training and recruitment they talked about may have happened some years ago.

**How will these findings help autistic adults now or in the future?**

Participants highlighted challenges and difficulties they face at work that affect their health and limit career progression. Findings show that employers and colleagues need better understanding of autism to reduce discrimination and help autistic professionals feel supported and safe to talk about their needs. This would help employers work with autistic professionals to identify changes to workplace habits and ways of working, which can support the well-being and career progression of autistic professionals.

## Background

There is a need for research exploring the experiences of autistic people who have been successfully employed on professional career paths.^1^ Increased understanding regarding these accomplishments can facilitate inclusion, enhance representation in work, and support improvements in the profile of autistic people in society.

Autistic people are underrepresented in the workforce and are more likely to experience environmental and cultural barriers, including discrimination and stigma.^2–4^ These factors exist at all levels of employment, with autistic graduates more likely to be unemployed than other graduates, including graduates with physical or mental health disabilities.^5,6^ Previous research has concentrated on young people yet to complete training, or adults who work in unskilled positions, and little is known about the career progression of autistic adults in competitive, skilled employment.^1,7^

The neurodiversity paradigm promotes the need for societal change to create welcoming supportive environments for autistic people.^8^ Autistic people often cite social communication and interaction as barriers to employment, alongside preferences for consistent routine and predictability, and sensory differences.^4^ Social differences and the “double empathy” problem, where there are breakdowns in communication and understanding between autistic and non-autistic people, present unique challenges at each stage of the employment process, including during job interviews and work interactions with supervisors and colleagues.^4,9–12^ Skilled or professional jobs often have additional barriers to employment, including clinical practice placements and competitive selection processes.^7^ Anecdotal reports from autistic professionals highlight work culture issues, including derogatory stereotypical views of autistic people and tokenism, which contribute to anxiety over disclosure and reluctance to seek support.^13^ This can lead to people choosing not to disclose their autism diagnosis and masking their identity to prevent stigma and discrimination,^14^ meaning that autistic professionals may be more prevalent than figures suggest.^15,16^

People working in health and education are particularly well-positioned to challenge societal preconceptions, positively influence social and physical environments, and model neuro-affirming language and interactions.^16,17^ Eighteen percent of autistic graduates work in the education sector making it the most frequent destination for autistic graduates.^5^ Autistic teachers have the potential to act as role models for learners and can facilitate inclusion through their own lived experience.^18,19^ They describe experiencing deeper understanding and being able to communicate more effectively with autistic learners.^20^ However, emerging research has indicated that autistic teachers face significant challenges, employers deny them professional opportunities, they avoid promotion, work reduced hours, experience mental health issues and fatigue, or leave the education sector altogether.^19,20^

Similarly, a diverse health care workforce that understands their patients’ needs by virtue of shared experience better serves a diverse population.^21^ Research has considered the experiences of autistic doctors, and while this is useful, they represent a small proportion of the health care workforce. Around 1.1% of psychiatrists, and 1% of general practitioners identify as autistic although most autistic doctors may remain undiagnosed or choose not to disclose their diagnosis.^14,22,23^ Employers seek characteristics such as attention to detail, memory, and visual processing, and autistic doctors also excel at task-focused and structured activities such as patient care.^21,24^ Autistic doctors report most misunderstandings arise during interactions with management, or regulatory bodies.^22^

Despite the potential for positive influence within these roles, particularly relating to the environment surrounding the care and nurturing of young autistic people, the broader experiences of autistic professionals working within the education and health care sectors remain underexplored.^18,25–27^

This study was conducted by members of the National Autism Implementation Team (NAIT), which is a multidisciplinary team funded by the Scottish Government focused on the development of public sector health and education services in Scotland for autistic and neurodivergent people within a neurodiversity informed paradigm.^17,28^ The Scottish Government as a leader and public sector employer is working to protect and promote the rights of neurodivergent people.^29,30^ The Scottish and UK Governments have identified reducing inequalities in employment for autistic people as a target for action.^29,31^ This requires understanding of the challenges facing neurodivergent people working in the public sector in Scotland.

The aims of this study were to explore the employment-related experiences of autistic people working in professional roles within health care and education in Scotland, to understand factors that affect access to professional careers and the subsequent career progression. We intended to identify practices and circumstances that support or challenge fair and inclusive work practices for autistic professionals working in the health and education sectors in Scotland.

## Methods

This study received approval from the Queen Margaret University Ethics Committee. All participants provided written informed consent before taking part in this study.

### Participants and recruitment

Purposively sampled participants were all 18 years or older, with a diagnosis of autism or who self-identified as autistic and were able to participate in an online interview in English. Participants were employed in a paid public sector role in health or education in Scotland requiring professional training, had full registration with a relevant statutory body (e.g., Health Care Professions Council-United Kingdom, General Medical Council, General Teaching Council for Scotland), and had been working in practice for at least 2 years.

Research team members circulated study recruitment information on social media, including Twitter (now X) accounts and by email to group contacts concerned with health and education in Scotland. Researchers invited interested persons who fulfilled the inclusion criteria to contact them. Participants received £50 gift vouchers in recognition of their contribution to the study.

We obtained data from participants (*N* = 34) aged between 24 and 57 (M = 43.41, SD = 10.24) years, who mainly identified as female (*n* = 22, 64.7%) and as White English, Scottish, Northern Irish, or British (*n* = 30, 88.2%). Participants received a formal diagnosis of autism from a health professional (*n* = 25, 73.5%) aged 5 to 56 (M = 36.96, SD = 12.62) years, while others were self-diagnosed (*n* = 9, 26.5%) (Table 1). Only two participants (6%) received an autism diagnosis during childhood. Most participants (*n* = 25, 73.5%) had disclosed their autistic identity to their employer.

**Table 1.** Participant Characteristics

Characteristic	*n*	%
Gender		
Male	10	29.4
Female	22	64.7
Nonbinary	2	5.9
Ethnicity		
White English, Scottish, Northern Irish, or British	30	88.0
White Irish	1	2.9
White—other	2	5.9
Mixed	1	2.9
Employment		
Full-time	28	82.4
Part-time	6	17.6
Highest Qualification		
Higher national diploma (HND)	1	2.9
Bachelor’s degree	17	50.0
Master’s degree	3	8.8
Postgraduate certificate	4	11.8
Postgraduate diploma	7	20.6
Doctoral degree	2	5.9
Diagnosis Method		
Formal diagnosis	25	73.5
Self-diagnosed	9	26.5

*Note*. *N* = 34.

Participants were employed in professional roles within education (*n* = 12, 35.3%) or health (*n* = 22, 64.7%) sectors across seven health board areas and eight local authority areas representing urban and remote rural areas of Scotland. Participants employed within the education sector included teachers in primary and secondary education with specialties, including computing science, physics, biology, modern languages, modern studies, music, inclusion and well-being, and support for learning. Participants employed within the health sector held a wide range of professional roles, including general practitioner, dietitian, podiatrist, clinical scientist, occupational therapist, specialist health promotion nurse, clinical nurse in psychological therapies, and radiographer (Table 2).

**Table 2.** Participant Roles

Identifier	Age	Gender	Role
EDU1	50	NB	Secondary teacher, science
EDU2	51	Male	Secondary teacher, languages and support for learning
EDU3	31	Female	Primary teacher
EDU4	37	Female	Secondary teacher, music and support for learning
EDU5	57	Female	Secondary teacher, languages and support for learning
EDU6	47	Female	Primary teacher, inclusion and well-being
EDU7	24	Female	Secondary teacher, science
EDU8	41	Female	Secondary teacher, science
EDU9	27	Female	Secondary teacher, science
EDU10	39	Male	Secondary teacher, computer science
EDU11	48	Female	Primary teacher, early years
EDU12	57	Female	Primary teacher
HEA1	54	Female	Clinical scientist
HEA2	52	Female	Occupational therapist
HEA3	50	Female	Mental health nurse
HEA4	41	Female	Clinical psychologist
HEA5	53	Female	Nurse
HEA6	29	Female	Health promotion nurse
HEA7	52	Male	Podiatrist
HEA8	48	Male	Adult nurse
HEA9	36	Male	General practitioner
HEA10	43	Female	Nurse
HEA11	33	Female	Speech and language therapy
HEA12	55	Female	Social worker
HEA13	51	Male	Counseling psychologist
HEA14	45	Male	Manager
HEA15	49	Male	Care manager
HEA16	54	NB	Clinical nurse specialist
HEA17	26	Male	Adult nurse
HEA18	25	Female	Dietitian
HEA19	46	Male	Health improvement officer
HEA20	55	Female	Nurse
HEA21	31	Female	Radiographer
HEA22	39	Female	Biomedical scientist

*Note. N* = 34.

EDU, education, HEA, health.

### Procedure

Before recruitment, an autistic coauthor working in a professional role piloted the interview process, and the interview was subsequently amended. Changes included asking participants to focus on their career rather than a typical day at work and to consider specific challenges or benefits related to being an autistic professional (Table 3).

**Table 3.** Interview Questions

1. About your training
1.1. Tell me what it is like being an autistic person in training—please think about when you decided that this is something you wanted to pursue—so this encompasses pretraining and your formal training—so what supported you personally, and what caused you problems?
1.2. Could you give me your views on how effective your educators and student colleagues were in helping you?
1.3. Did you disclose and if so to whom? *(Could I ask you to explain to me why you did or did not disclose? What were the consequences of this?)Optional Prompt: Is there anything else from your training time that you think is important and you would like to discuss? *
2. About recruitment
2.1. Tell me what it is like being an autistic person job seeking and going through recruitment—What factors MOST supported you personally, what caused you MOST problems?
2.2. Did you disclose and if so to whom? Could I ask you to explain to me why did you or did not disclose? What were the consequences of this? *Optional prompt: Are there any other issues from the recruitment time that you think are particularly pertinent or you would like to discuss?*
3. Your current job
3.1. Again, what we are interested in here is your experience at work. As best as you can make out, tell us what it is like being an autistic person in your current job and what has supported you, and what has caused you problems?*Optional prompt: What do you enjoy about your job? What things are you good at and enjoy the most? Is there anything that you struggle with particularly?*
3.2. Did you disclose and if so to whom? (*Could I ask you to explain to me why did you or did not disclose? What were the consequences of this?)*
3.3. What has supported or restricted your career success? We are interested in this in relation to your autism, and how that has influenced things. How did you get to where you are now in your career *(how did you manage the pressures and stresses?) (and WHERE RELEVANT/for more senior people, specifically with regard to career progression?)Optional prompt: Where does your support come from?*
3.4. Overall, could you tell me how the physical or sensory environment contributes to your experiences at work? Again, in relation to your experiences as an autistic person.
3.5. Overall, could you tell me how the social or cultural environment contributes to your experiences at work? Again, in relation to your experiences as an autistic person.
3.6. Tell me about whether your identities as both an autistic person and as a professional sit easily together, or do you ever find them in conflict?(Possible prompts e.g., *masking, or when you are involved in providing support or training on autism? Or your lived experience being at odds with things that you see in your work, or things that people say about autism at work?*)
4. Your Recommendations
4.1. Please think about your training experience—what are your top recommendations for changes or improvements that could be implemented to help autistic trainees?
4.2. Please think about your overall career—what advice would you give to an autistic person wishing to pursue a career like yours? Guidance about how autistic people can successfully progress and how they can find a rewarding career in their profession?
4.3. Please think about things you would really like to see changed in work culture for autistic people—what are the top things workplaces could do to support autistic people?*Optional prompt: If you had free choice, what would you change in the working environment?*
4.4. Lastly, if you were to design autism training for organizations or professionals, what would be the most important things to include in this training?*Optional prompt: what would you like other people to understand about autism?*

The research team asked potential participants to complete a brief questionnaire that collected contact information and confirmed that they met the inclusion criteria. Potential participants who met the inclusion criteria received study information, including interview questions, and consent form. The study information pack included contact details for the research team in case of questions relating to participation in the study, and details of counseling, health, and social support agencies in case participants required assistance. Following return of a signed consent form and completed demographic information form, researchers invited participants to take part in a semistructured interview conducted online using MS Teams.

Seven members of the research team conducted interviews (E.C., D.M., L.J., M.M., V.J.-C., I.U., and M.R.). Before taking part, we asked participants if they had any concerns about participating, or any questions relating to the information contained in the consent form or study information pack. Interviewers advised participants that they were welcome to take breaks as required during the interview or to leave the interview at any time; and asked how they would indicate when they required a break. The interviewer asked the interview questions verbally and typed them into the chat section of MS Teams.^32,33^ Interviews typically lasted 60 to 90 minutes and included open questions regarding participants’ firsthand experiences of training, recruitment, sensory environments, their current job, with reference to their autism, and to provide recommendations that could enhance support (Table 3). We recorded interviews electronically and had them professionally transcribed. During data collection, researchers met regularly to reflect on personal insights noted during the interviews, observed data patterns, and potential codes.

### Data analysis

The first author (E.C.) conducted a reflexive thematic analysis, using a data-driven inductive approach.^34,35^ Researchers uploaded interview transcripts to NVivo for analysis.^36^ The first author (E.C.) immersed themselves in the data, reading and rereading all transcripts closely, noting recurring or standout observations and informal reflections. E.C. then read transcripts line-by-line and organized data into common groups or codes. EC applied codes throughout each transcript. We applied a “complete coding” approach and gave each unit of text equal consideration; some sections of data were assigned to multiple codes, while others were not coded.^34^ Codes were then organized into groups based on their shared meaning by E.C., and then refined to create themes. We developed a thematic map to visualize the relationships between themes and subthemes. Research team members (E.C., D.M., V.J.-C., and M.M.) regularly discussed the developing themes to ensure they made sense, and to establish depth and robustness.^35^ The first author developed a report that reflected the themes in a cohesive, meaningful narrative. We defined themes and subthemes by their unique characteristics and supported by examples from the data. D.M. and E.C. finalized a narrative presentation of the thematic analysis, and the whole team reviewed and refined the themes and subthemes to ensure coherence and to provide additional insights.^34^

### Author positionality

The principal investigator for the study (E.C.) is not autistic and is an occupational therapist and research fellow with NAIT, conducted most of the interviews, led the analysis, and coded all the transcripts. All authors are associated with NAIT, a team that includes autistic and non-autistic people, although they are not all disclosed. Team members are clinicians (psychologists, occupational therapists, speech and language therapists, psychiatrists), researchers, and educators who have experience working with autistic people. An autistic coauthor identified the topic as important and codeveloped the study protocol and research questions.

This article followed the Standards for Reporting Qualitative Research guidelines.^37^

## Results

**FIG. 1.**
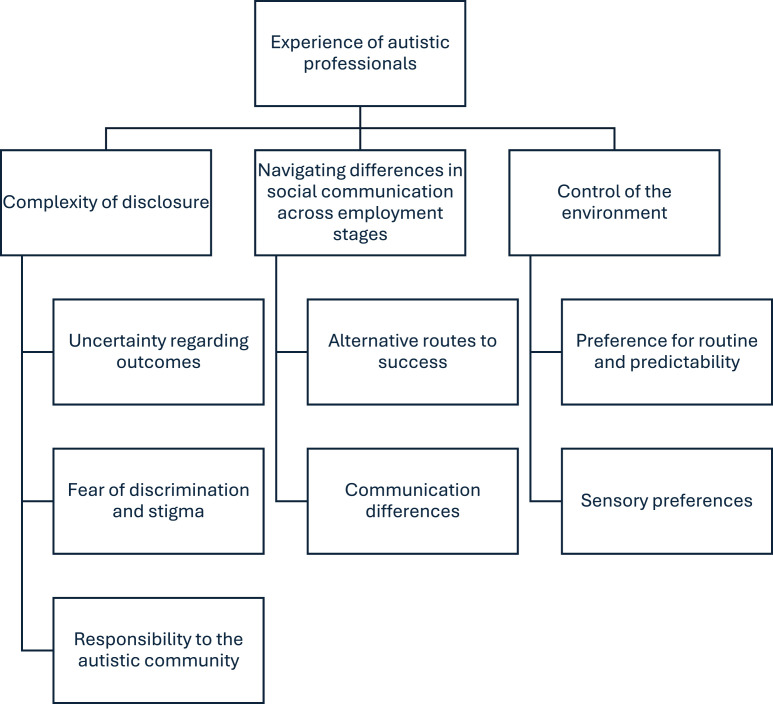
Thematic map.

We included a thematic map showing the final set of primary themes and subthemes about the employment experiences of autistic health and education professionals in Figure 1.

We produced three dominant themes from the interview data as follows: (1) complexity of disclosure; (2) navigating differences in social communication across employment stages; and (3) control of the environment. We labeled quotations to indicate if the participant worked in health (HEA) or education (EDU) (Table 2). We removed filler words, but otherwise reproduced quotations verbatim, and therefore they may contain grammatical errors and individual’s language choices.

### Theme 1: Complexity of disclosure

Theme 1 examines the intricate and burdensome decisions participants made regarding the disclosure of their autistic identity at work. They used carefully considered strategies to achieve workplace adaptations and support and sometimes concealed personal information to reduce exposure to negative impacts.

The decision to disclose an autistic identity was complex, and an area of considerable reflection for participants. Most participants had shared aspects of their identity at work but deliberated carefully about what and to whom they disclosed personal information. Sometimes, participants felt obliged to disclose to their manager or to human resources (HR) such as one participant who, “*chose just to disclose to the head of department*” (HEA9). Another participant preferred to disclose to people she knew well, *“I don’t tell people until I trust them as a person” (EDU3)*. Following negative reactions to disclosure, one participant chose not to share this information at all, “*What I realize now is that I don’t have to disclose*” (EDU7).

Participants were less likely to disclose if they did not have a formal diagnosis, did not have a close relationship with the person, felt it would provide no benefit, or may subject them to discrimination. “*If they don’t ask, I won’t tell them*” (EDU2). Where participants had co-occurring conditions, they found they could disclose an alternative diagnosis such as dyslexia, to access adjustments they required without attracting the negative consequences they sometimes experienced following autism disclosure. Another participant preferred selection for interview based on her skills and experience, “*I want to get an interview because my application’s good, not because I have a disability*” (HEA11).

#### Uncertainty regarding outcomes

Disclosure did not always lead to the expected response. Participants found that colleagues could be supportive and offer helpful advice, “*The person from occupational health was lovely, she listened to all of my concerns, […] and I had chatted her through reasonable adjustments I needed*” (HEA11). However, this experience was not universal and sharing personal information could also result in discrimination, resulting in participants being more reluctant to share personal information.

For some participants, disclosure was a negative experience that they didn’t wish to repeat as it led to negative outcomes such as losing out on work, “*I shouldn’t have disclosed in the past because I’ve had adverse effects where I’ve lost out on work because I did disclose*” (HEA6). In addition, it was often not a one-off event, and due to staff turnover, participants described having to discuss their autism repeatedly,

So, I have to just tell them every time. I just have to go through it every time [and it] is the most depressing thing ever and it puts me in such a horrible mood and having to tell people over and over again what I have problems with because it’s the problems we need to address. It’s not oh by the way, I’m really good at learning all this information. By the way, I’ll remember this (HEA22).

Participants found that their expectations regarding support and workplace accommodations were often unfulfilled leaving them confused and unsure about how to proceed. HR and management were sometimes unaware of actions they could take to support autistic employees, or were unwilling to consider these,

I told the management team from the outset that I was autistic after I’d been offered the job. I’m not sure what I expected to happen, but nothing happened. It was never brought up again. They didn’t ask about it. They didn’t ask if there was anything that I needed help with and I didn’t really have the confidence to go and ask for help (EDU3).

Other consequences were also apparent, including recruiters supplementing recruitment processes. One participant (EDU7) described how she lost out on a job opportunity as she disclosed her autism diagnosis on the application form. Recruiters then required a satisfactory medical review before awarding the employee the job. The review took months to arrange, meanwhile recruiters filled the post with another candidate.

#### Fear of discrimination and stigma

Participants found disclosing their autistic identity to colleagues or managers could lead to exclusion and discrimination,

No, it wasn’t a positive thing unfortunately and I didn’t really get much understanding and genuine support. I think it was almost – I could feel sometimes that – I sort of regretted sometimes being so open and honest because I felt it was almost used against me so sadly not a positive situation (HEA7)

and

I know that some people have thought well, how can you be a teacher if you’re autistic (EDU3).

An employer offered one participant a promoted post, based on their success. However, once the participant shared their autistic identity, the manager reversed this decision,

I was handpicked by the head of nursing for this post, she’d scoped me out because she knew from COVID and from the ward I worked on, that – like I used to run the ward myself when the senior charge nurse wasn’t in. It was something that sort of came naturally to me and I could keep everyone calm and if there was a crisis, I’d be fine, I could get it under control. So, she kind of handpicked me and then for me to say that I had autism and for her to drop me, it put a dent in the way I felt about my job and how I was. I thought I’m not a good nurse now (HEA6).

Participants adopted practices to prevent colleagues identifying them as autistic, “*I’m pretty bloody good at just pretending to be like everybody else*” (HEA4). One participant even shared with colleagues that they adopted a workplace persona, “*I do say to people, what you see at work is not what I am at home. Just so that if they met me out in the street they wouldn’t be surprised if I just said hello and walked by them” (*HEA20).

Teacher colleagues were understanding of the needs of autistic learners, but participants felt they did not receive similar support,

I think in that respect a lot of people, I think they think ASD children drop off the end of the earth when they grow up and then they suddenly flick a switch and they become that person coming into the workplace that they don’t quite like, that’s a bit weird. I just really want to educate them with that, that the next time a weird person is in your workplace, consider they might have been an ASD kid and that way they could relate to them more (EDU12).

One manager referred a participant for additional training as they completed tasks in an unusual way compared with neurotypical colleagues. They were frustrated by their manager’s actions, which they felt suggested they lacked competence,

Let’s think about what my limitations in the context of needing training on a particular subject. Why do I need training on that subject? Is it your perceptions of me and what I’m doing and how I work? Which might actually, if you understand a bit more about that, you understand that I don’t lack skills in that, it’s more about your appreciation of how I do things (HEA14).

#### Responsibility to the autistic community

Participants reported that one of the reasons they disclosed was because they viewed this as a route to raising awareness of autism, to increase wider acceptance and understanding,

All the staff are aware because I’ve disclosed to them that I’ve got autism. Also, the pupils as well, I’ve disclosed to them and that’s actually worked quite well because a lot of them have seen me as a good role model (EDU7).

Participants said that their personal experience gave them an advantage over non-autistic colleagues to support autistic learners. “*I have an understanding of the kids that I work with that a lot of people don’t have*” (EDU3). Lived experience of autism made participants more understanding of people with similar characteristics and increased their empathy. Participants reasoned that they should use their experience for the benefit of others,

One of the reasons I wanted to become a schoolteacher in the first place even before an autism diagnosis is because I had really bad experiences at school, and I didn’t want other people to go through the sort of education that I had (EDU2).

Although as the experts in this field, managers often assigned participants additional tasks such as providing relevant training for other staff or attending to neurodivergent patients, *“When we have autistic patients come in, I think I’m definitely the person that people call on to come and talk to them”* (HEA10).

Younger colleagues were often more accepting of their autistic colleagues, perhaps because people are now more open about autism:

We’ve got a lot of younger staff, and I think they’ve come up through school knowing a little bit more about neurodiversity. There’s probably an awful lot more of them actually have a diagnosis and they’re really cool with it (HEA23).

Other participants found themselves working within teams of like-minded people, “*we’re all quite kind of quirky characters*” (HEA5), and “*it’s a very inclusive team I’m in, from mental health, physical health and neurodiversities, the vast majority of us are fully disclosed*” (HEA8). Participants thrived when employed in departments that valued neurodivergence and reported positive benefits from working with autistic children, or in health teams with responsibilities for the support of neurodivergent people.

### Theme 2: Navigating differences in social communication across employment stages

Theme 2 considers how differences in communication styles between autistic and non-autistic individuals in the workplace can lead to misunderstandings and difficulties. Participants highlighted instances where colleagues’ language or cues were perplexing, making it challenging to understand each other. Such issues extended to interview scenarios, where interpreting questions posed an extra hurdle, meaning that securing employment required alternative strategies. The theme further illustrates the intricacy of hidden social rules and expectations, which often lead participants to stress and uncertainty. While guidance from colleagues or partners could be helpful, there remained instances of insensitivity and misjudgment.

#### Alternative routes to success

Participants described challenges associated with gaining employment or promotion at work due to limited success in interviews. They described difficulties with understanding what interview questions sought to elicit, *“I can remember really struggling, and actually (I’m not kidding you), going for interview after interview, and just not having a scooby [clue] what the game was, and being mystified by it” (*EDU1) or “*it feels like an exam that you’re not allowed to give the right answer […] only the one they want to hear*” (HEA13).

This meant that a few participants had never been successful at an interview but managed to obtain employment or promotion through alternative means such as in-post promotions or by accepting locum positions where they were able to demonstrate their capabilities, “*The school that I got my first job at had seen me in the classroom, so they knew that I was good at it*” (EDU3).

Another participant described extensive work they undertook to develop interview skills, including applying for jobs before they were ready for promotion. This provided opportunities to request feedback when they were unsuccessful in the recruitment process. They gradually became more comfortable with the interview process, adjusting their presentation and responses, and were able to predict questions that were likely to arise.

#### Communication differences

Participants provided examples of differences in the ways that they and their non-autistic colleagues communicate, which lead to difficulties in understanding work practices and social situations.

I think some of my colleagues think, oh, that’s a bit of an odd way to do it. Whereas I hear them and think, oh, I don’t have a clue what you’re talking about, with this wiffley-waffley language (HEA3).

Participants gave examples of missing nonverbal indicators, *“Will I recognise the cues when to shut up? No, I won’t”* (HEA14), and struggles such as *“understanding sometimes people’s humour and sarcasm”* (HEA17). There were also incidences, where colleagues interpreted failing to follow unspoken rules as rudeness, *“I’ve had incidents like that where colleagues that I’ve just met, we’ll have a conversation and I’ll think, that’s the conversation ended, I’ll just walk away without saying anything” (HEA16).* Colleagues could support understanding by using alternative communication methods, “*she’d be like, I’m going to draw it, and I didn’t realise that was a real factor in helping me until she did that*” (HEA11).

Participants expressed concerns regarding the navigation of social situations, “*I sometimes get the wrong meaning with things and vagueness is terrible*” (HEA7). Hidden curriculums or unspoken social rules added to the stress and unpredictability of work-related situations, “*I sometimes ask one of my colleagues to review an email, for example, just to make sure, how do I describe it, I say ‘can you translate humans for me?’*” (HEA8). For example, colleagues did not specifically name one participant in a work group chat, so the participant incorrectly assumed their colleagues had not invited them to a work event,

Participants appreciated having trusted colleagues and partners who could provide interpretations of unspoken social rules:

The only time I have struggled in work is, office etiquette I didn’t realise was a thing, nobody ever told me so I would go and get myself a cup of tea and not ask. I’d come home and say to my partner, and she was like, right, we need to sit down, and this is actually how an office works. Like if you’re making tea, you ask if everyone wants tea (HEA6).

### Theme 3: Control of the environment

This theme considers how challenges associated with unpredictable aspects of the physical and social environment contributed to participant’ stress. Participants excelled in structured protocol-driven environments. However, unexpected or uncertain situations in unfamiliar settings caused anxiety. Unmet sensory needs or preferences also impacted efficacy and well-being. Participants described strategies, including planning and scripting, but were often dependent on the consideration of colleagues, which was not always forthcoming. Cumulative stressors could result in burnout often meaning time away from work. There was a need for increased awareness and flexibility regarding adaptations in the workplace.

#### Preference for routine and predictability

Participants selected careers where autistic characteristics helped them excel at work, “*I’m very analytical and really good at noticing details*” (HEA22). They appreciated working in areas where protocols and procedures determined appropriate reactions to specific situations, supporting their preferences for routine and predictability.

I think in my current job, the things that I’ve actually managed really well is actually the clinical side of things because everything is logical in intensive care. Most things are protocol driven and they’re very clear protocols (HEA5).

However, they found situations that were undetermined, ambiguous, or unplanned in unfamiliar environments, including social events, interviews, timetable changes, and practice placements, or teaching methods such as role-play, to be difficult and stressful. One participant prepared extensive scripts in advance of teaching classes, “*I had everything written out because I didn’t know how to interact with a group of people*” (EDU4). Another participant who was usually comfortable in professional situations found interview situations incredibly stressful. “*I walk into the room and because I have no control over what they’re going to ask me, there’s no preparation, I just completely lose it*” (HEA5). Providing information in advance, such as the interview questions, meeting agendas, and protocols, could alleviate the impact of some situations.

#### Sensory preferences

Participants described challenges in tolerating aspects of the environment, including lighting, noise, temperature, touch, reorganization, and limited personal space,

Suddenly to go into a building, where it’s like, everything is fluorescent and LED. It’s on all the time, ugh – and its teeny windows, and the room is either freezing or boiling, and there’s nothing in between – as is the way of the NHS – was harder than I ever realised (HEA4).

Where these elements were outside a comfortable range, participants reported effects, including feelings of panic and reduced concentration.

For example, if we’re doing handover and there’s people – now to most people, they’ll sit and do handover and it doesn’t bother them but, to me, I can hear everything round about. I can hear a bag rustle, hear somebody cough, hear the monitor, hear the people next to me chattering and all those noises are really amplified for me. So, I find it really difficult to focus on what I’m being told because I’m too distracted by the environment (HEA5).

Other obstacles, including inflexible room booking systems and classroom allocation traditions, prevented participants from accessing work areas better matched to their sensory requirements. Colleagues interpreted participants’ coping strategies as rudeness, “*If I was really, really stressed or trying to figure something out, I go mute because I need to focus, I need to block the noise out, and to people, that comes across as just being angry and rude*” (HEA5).

The cumulative effect of stressors could affect well-being and lead to autistic burnout,

For me there’s physical and there’s like emotional meltdowns and the emotional ones I’ve generally got control of my body but when I’m physically overloaded, its more or less a seizure. I’m on the floor, I can’t move, I can’t move for hours. Everything just sort of shuts down (HEA22).

Participants also described long-term major impact on their mental well-being,

I’m on permanent antidepressants, and when things get too much, I just take a holiday for a week or two (HEA1).

and

I’ve had a lot of issues with self-harm and things like that at home at the end of the day and that I think is a lot to do with sensory build up that I experience over the course of the day at school (EDU3).

Some coworkers made adjustments when they noticed their autistic colleagues struggling, such as closing doors to exclude noise and using small desk lamps rather than fluorescent overhead lamps. Mostly though, participants developed their own regulation strategies, which included seeking flexible working arrangements such as working from home, negotiating with janitors for lower level lighting in hallways, adopting out-of-hours roles so they could work when the office was quieter, teaching subjects with smaller class sizes, and offering excuses to avoid busy staffrooms or social events. “*I tend to go into school much earlier than what I legally or contractually need to, because its lovely and quiet*” (EDU1). Participants working in promoted roles were able to specify environmental arrangements and working practices aligned with their own preferences, “*they like the routine that I give them, and I like the fact that they work to my routine*” (HEA20).

## Discussion

Neuro-affirming practice aims to preserve autistic ways of being and “my authentic self” while supporting well-being.^38^ This requires workplaces to accommodate naturally occurring variability and be cognizant of the perspectives and experiences of neurodivergent people.^8,17^ This study explored the experiences of autistic adults working in professional roles within health and education in Scotland. The participants in this study are successful, having secured employment in intensive and competitive professional fields, including teaching, medicine, nursing, and the allied health professions. However, despite their success, all participants described encountering significant ongoing challenges within the workplace.

This study confirmed that stereotypical views of autistic people are present within health and education workplaces.^20,39^ Professionals working in health and education have a key role to play in supporting autistic people to develop strong autistic identities and to be able to identify what they need to live well and maintain successful employment. However, while professionals may be supportive of autistic patients and learners, participants in this study reported that they appeared less understanding of the needs of neurodivergent colleagues. As in previous research, participants reported instances of individual and institutional ableism, including bullying, which discouraged them from disclosing needs, and from accessing required workplace accommodations.^40,41^ This marginalization and devaluing of neurodivergent colleagues may be rooted in stereotypical views about the capabilities required by professionals to deliver care safely.^40,41^ Policies and procedures require review to ensure they are neuroinclusive and do not support ableist agendas, which limit the participation and progression of disabled people in professional training and employment.^42,43^

Disclosure was a complex issue. Some participants reported positive experiences following disclosure, where they felt surrounded by colleagues who appreciated them for who they were. Such colleagues took responsibility for initiating changes that provided support for neurodivergent team members such as offering quiet spaces for them to work, adjusting office lighting, and flexible working arrangements. In other cases, disclosure was difficult and led to experiences of exclusion and discrimination causing participants to feel shameful about their identity.^14,44^ Participants had to carefully negotiate disclosing support needs to colleagues to access assistance, while maintaining their professional identity and social relationships.^45^ This included adopting masking strategies to hide aspects of their identity from colleagues, which is known to negatively impact mental health.^46,47^

UK employers are bound by law to make reasonable adjustments when they know or could reasonably know an employee is disabled.^48^ In line with previous research,^49^ we found that organizations did not always offer reasonable adjustments, or implement agreed adjustments, or maintain adjustments in the longer term. This was possibly because of limited awareness about autism and which adjustments may be helpful together with difficulties in making such requests, but research needs to confirm this. Sometimes, requesting adjustments resulted in unfavorable attention, particularly during recruitment processes. People should be confident that requesting adjustments will not affect their work status, job security, or chance of promotion.^50,51^ Employers should maintain adjustments for as long as the employee requires them even if the person moves to another work role, or if the adjustments no longer appear useful.

Our findings demonstrate a need to normalize requests for reasonable adjustments and to ensure that organizations carefully consider these requests and prioritize the well-being of the individual.^43,52^ Rather than remediating deficits, workplaces should focus on removing barriers and designing accessible processes,^53^ such as offering adjustments to all employees without the need to declare a protected characteristic, perhaps as part of an annual review process.^31,43^ Such antidiscriminatory or neuroinclusive practices increase accessibility to adjustments and reduce stigma.^49,54,55^ Participants urged organizations to offer adjustments during the recruitment process as they found it most challenging to make requests when they were unfamiliar with the workplace culture. This also establishes the organization as being supportive of neurodivergent people.^52^ Researchers can help by identifying adjustments that people working in similar roles have found beneficial for consideration by autistic employees and their managers.^6^ Many of the adjustments that autistic people request cost nothing and only require a change of mindset.^56^ Using a framework such as Autistic SPACE (Sensory needs, Predictability, Acceptance, Communication, and Empathy) could provide a foundation for training and conversations around possible areas for adjustment.^56^

Participants reported that they appreciated colleagues or friends interpreting social nuances for them. However, people must ensure that they do not pressure neurodivergent colleagues to conform with social expectations or work cultures, which do not align with their own preferences. Creating a workplace that is accepting of individual needs, open to changes that meet these needs, and embraces diverse perspectives empowers people to feel accepted and able to be their authentic selves.^14,46^ This, in turn, will support the well-being of autistic employees reducing stress-related absence.^57^ Research suggests that workplace accommodations may benefit all employees^49^ as diverse teams or organizations may perform better than more homogenous groups.^58^ In addition, this will increase the visibility of autistic people, influence the disclosure of others, and support workforce diversity through improved recruitment, retention, and promotion of autistic people within the workforce.^31,40^ These outcomes will demonstrate the benefits of positive change, equal development opportunities, and inclusive work processes.^31,43,58^ Flexibility within recruitment and career progression can tailor processes toward skills essential for the job rather than focusing on social skills and presentation.^3,52^ Findings from this study confirm that there is a need to focus on characteristics relevant to the effectiveness of employees in their job, which may be better assessed through practical demonstrations than interview.^59^ A more flexible approach to other aspects of the work environment may also maximize productivity and job satisfaction.^3^

The participants in this study called for workplace training to develop colleagues’ understanding of autism and other neurodivergence, using lived experience examples. Participants anticipated that training would assist colleagues to become familiar with some of the characteristics of autistic coworkers and appreciate how changes, such as adapting their communication styles, could offer support.^60^ Training can be helpful when offered as part of a package of support,^61^ such as an autism tsar or champion to challenge areas of exclusion within organizations, and employee resource groups and support networks.^31,62^ These groups can also hold organizations to account when they fail to implement relevant legislation or policy.^62^ Coproduction of workplace policies and procedures with representatives of minoritized groups can also promote inclusivity.

### Limitations

The study sample mainly identified as White and female. The population in this study had a higher proportion of people who identified as White than indicated in recent Scottish census numbers.^63^ Participants included self-identified and formally diagnosed autistic people, and the research team did not confirm their autism diagnosis. The study sought views and experiences regarding their training and recruitment experiences, however, these experiences may have occurred years in the past.

## Conclusions

There is a strong need for change of work culture within Scotland’s public sector. This study confirms that neurodivergent health and education professionals face institutional and individual ableism during training and in the workplace.^7^ Inflexible processes, discriminatory attitudes, and failure to provide and maintain workplace accommodations are major challenges for autistic professionals. The Scottish Government has committed to embedding fair work practices across the public sector, including reducing the disability employment gap and improving labor market outcomes for minoritized people.^30^ To achieve this, there is a critical need to remove barriers around the training, recruitment, and employment of neurodivergent health and education professionals.
